# Targeting cGAS-STING pathway for reprogramming tumor-associated macrophages to enhance anti-tumor immunotherapy

**DOI:** 10.1186/s40364-025-00750-w

**Published:** 2025-03-12

**Authors:** Weiyue Zhang, Xin Huang

**Affiliations:** 1 Department of Endocrinology, Union Hospital, Tongji Medical College, Huazhong University of Science and Technology, Wuhan, 430022 China; 2 Department of Orthopaedics, Union Hospital, Tongji Medical College, Huazhong University of Science and Technology, Wuhan, 430022 China

**Keywords:** cGAS-STING, Tumor-associated macrophages, Tumor microenvironment, Tumor, Immunotherapy

## Abstract

The cyclic GMP-AMP synthase (cGAS)-stimulator interferon genes (STING) signaling pathway plays a crucial role in activating innate and specific immunity in anti-tumor immunotherapy. As the major infiltrating cells in the tumor microenvironment (TME), tumor-associated macrophages (TAMs) could be polarized into either anti-tumor M1 or pro-tumor M2 types based on various stimuli. Accordingly, targeted reprogramming TAMs to restore immune balance shows promise as an effective anti-tumor strategy. In this review, we aim to target cGAS-STING pathway for reprogramming TAMs to enhance anti-tumor immunotherapy. We investigated the double-edged sword effects of cGAS-STING in regulating TME. The regulative roles of cGAS-STING pathway in TAMs and its impact on the TME were further revealed. More importantly, several strategies of targeting cGAS-STING for reprogramming TAMs were designed for enhancing anti-tumor immunotherapy. Taken together, targeting cGAS-STING pathway for reprogramming TAMs in TME might be a promising strategy to enhance anti-tumor immunotherapy.

## Introduction

Anti-tumor treatment has always posed a significant challenge in the world. Nowadays, various anti-tumor therapies have been explored including surgery, radiation therapy, chemotherapy, immunotherapy [1]. Owing to the rapid advancements in immunology and deeper understanding of tumor microenvironment (TME), the significance of anti-tumor immunotherapy is now widely acknowledged [2–5]. Apart from the specific immune response driven by T cells, the role of innate immunity cannot be underestimated in anti-tumor immunotherapy [6]. It could detect the pathogen-associated molecular patterns, and further activate downstream signaling pathways, thereby promoting the release of interferons, cytokines, and other signaling molecules for anti-tumor effects [7, 8]. Accordingly, the pivotal role of innate immunity in anti-tumor immunotherapy is warranted to be uncovered.

Tumor-associated macrophages (TAMs) play a critical role in tumor immunotherapy, influencing both innate and specific immunity. Their presence is significantly associated with tumor development and a poorer prognosis of tumor patients [9, 10]. TAMs are highly adaptable, shifting between the M1 phenotype, which inhibit tumors, and the M2 phenotype, which promote tumor growth [11]. As the major infiltrating cells in TME, TAMs could be polarized into either anti-tumor M1 or pro-tumor M2 types based on various stimuli [12]. Accordingly, targeted manipulation of TAMs to restore immune balance shows promise as an effective anti-tumor strategy [13]. Current TAM-focused immunotherapy approaches include depleting TAMs from the TME and modulating their phenotype to treat tumors with low immunogenicity [14, 15]. However, the outcomes of these two therapies in anti-tumor treatment are still not satisfactory.

**Fig. 1:**
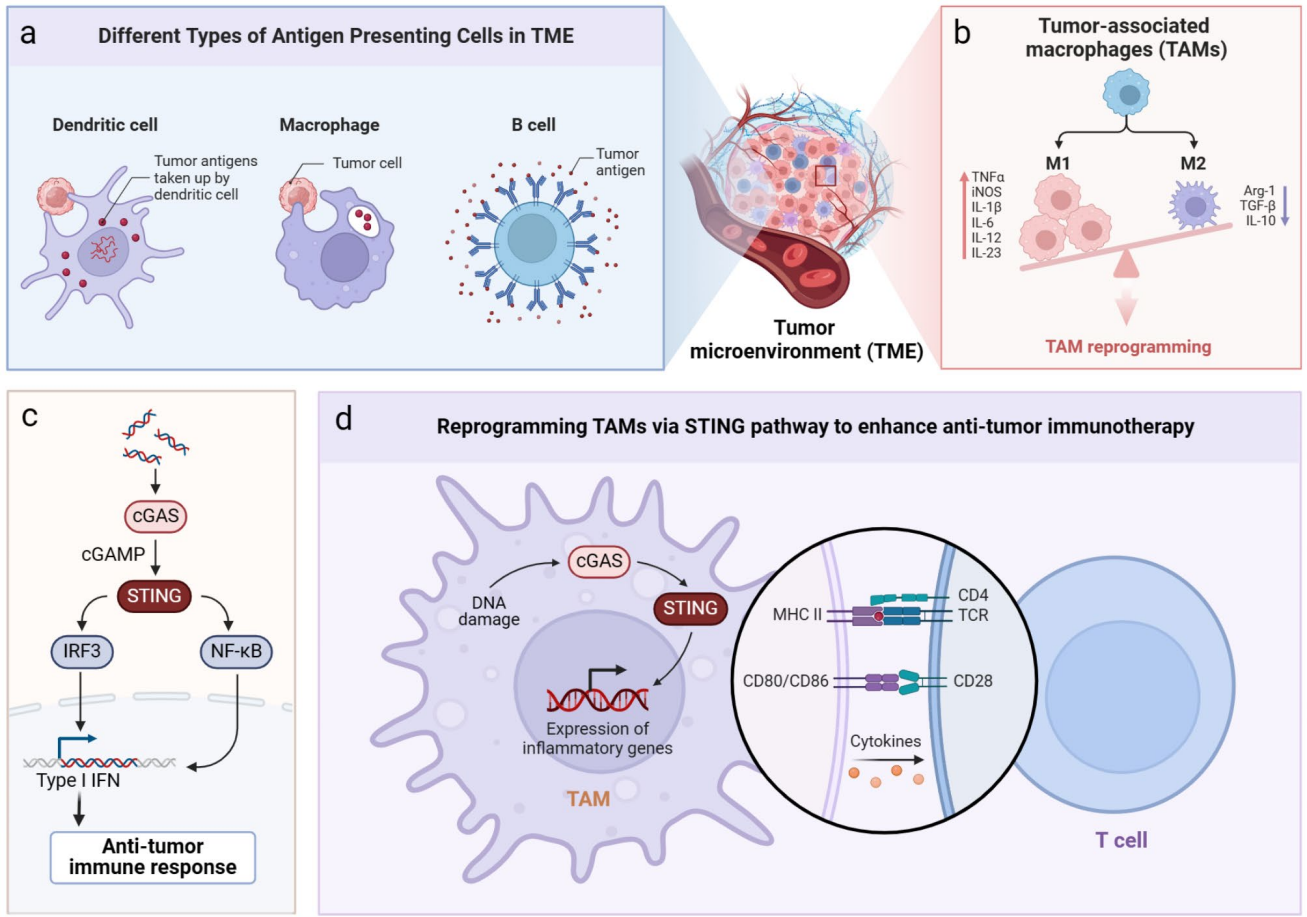
Reprogramming TAMs via cGAS-STING pathway to enhance anti-tumor immunotherapy. (**a**) The most common antigen presenting cells in the TME including dendritic cells, macrophages, and B cells; (**b**) As the major antigen presenting cells in the TME, TAMs could be polarized into either anti-tumor M1 or pro-tumor M2 types based on various stimuli. Reprogramming TAMs to restore immune balance shows promise as an effective anti-tumor strategy; (**c**) As a sensor for cytosolic DNA, cGAS could catalyze the synthesis of cGAMP to activate STING. Activated STING leads to the recruitment and phosphorylation of IRF3, and facilitates its translocation into cell nucleus where it participates in the transcription of type 1 IFN. The cGAS-STING activation could induce the anti-tumor immune response; (**d**) Reprogramming TAMs via cGAS-STING pathway could induce the anti-tumor immune response and further enhance anti-tumor immunotherapy

The cyclic GMP-AMP synthase (cGAS)-stimulator interferon genes (STING) signaling pathway is pivotal in activating both innate and specific immunity within anti-tumor immunotherapy [16, 17]. Monocytes, particularly macrophages, serve as the major source of type 1 IFN production through this pathway in human body [18]. Thus, investigating the roles of cGAS-STING signaling in TAMs and its effects on the TME are of great importance. This review aims to target the cGAS-STING pathway to reprogram TAMs, thereby enhancing anti-tumor immunotherapy (Fig. 1).

## The complex roles of cGAS-STING pathway in tumors

**Fig. 2:**
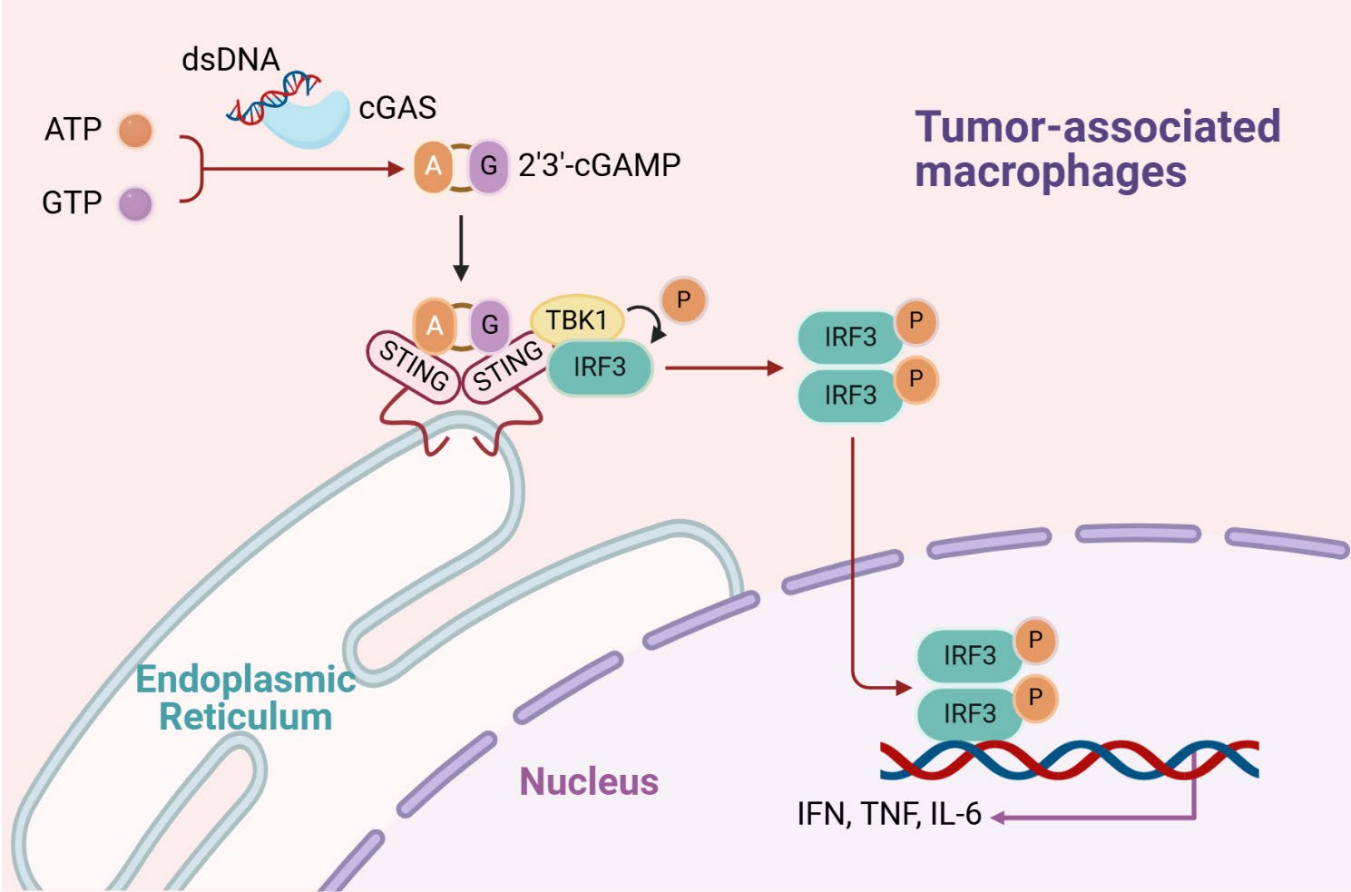
The cGAS-STING pathway. cGAS is activated by specifically recognizing free cytoplasmic DNA from itself and foreign sources, then uses ATP and GTP as raw materials to synthesize cGAMP, which binds to STING protein on endoplasmic reticulum as a stimulus signal to induce its conformational change and activation. And then the activated STING migrates from endoplasmic reticulum to Golgi apparatus, recruit and activate TBK1 and IKK kinases, thereby activating downstream IRF3 signaling pathways, ultimately inducing the expression of IFN and other cytokines, and enhancing the immune response

As a sensor for cytosolic DNA, cGAS has the ability to directly detect double-stranded DNA (dsDNA) within the cytoplasm. It could catalyze the synthesis of cyclic GMP-AMP (cGAMP) to further activate STING. Activated STING recruits TANK-binding kinase 1 (TBK1) and undergoes autophosphorylation, which could result in the recruitment and phosphorylation of interferon regulatory factor 3 (IRF3), facilitating its translocation into cell nucleus where it participates in the transcription of type 1 interferon (IFN) (Fig. 2; Table 1) [19, 20]. Given the genomic instability or oxidative stress observed in tumor cells, DNA leakage frequently occurs, triggering the activation of the cGAS-STING within the cytoplasm. The cGAS-STING could generate IFN, promote the maturation and migration of dendritic cells (DCs), activate TAMs and natural killer (NK) cells, and induce the expression of chemokines for the infiltration of cytotoxic T lymphocytes (CTLs). The above processes induce the anti-tumor immune response [21, 22].

**Table 1:** cGAS-STING pathway members and their functions and roles

Component	Activator	Function	Role in Immunity
**cGAS**	Cytosolic dsDNA	Senses cytosolic DNA, synthesizes cGAMP, activates STING	DNA sensor, initiates innate immune response
**STING**	cGAMP, bacterial CDNs	Activates downstream signaling upon cGAMP binding; Activates TBK1, IRF3; induces IFN-I and cytokines	Adapter protein linking DNA sensing to IFN response
**TBK1**	Activated STING	Phosphorylates IRF3, STING, and NF-κB	Transduces signal to transcription factors
**IRF3**	TBK1	Drives type I IFN gene expression; Induces IFN-α/β and antiviral genes	Key transcription factor for antiviral genes
**NF-κB**	TBK1	Regulates inflammatory cytokines, promotes TNF-α, IL-6, and other cytokines	Inflammatory response
**cGAMP**	DNA recognition by cGAS	Second messenger generated by cGAS, triggers STING activation	Activates STING
**DExD/H-box helicases (DDX41**,** DHX9)**	Cytosolic DNA or nucleic acids	DNA sensors that enhance cGAS-STING signaling and promote IFN activation	Enhance immune sensing and response
**TREX1**	Excess cytosolic DNA	Degrades cytosolic DNA, limits cGAS activation, and prevents chronic inflammation	Prevents autoimmunity

**Abbreviations:** cyclic GMP-AMP synthase, cGAS; stimulator interferon genes, STING; TANK-binding kinase 1, TBK1; interferon regulatory factor 3, IRF3; dsDNA, double-stranded DNA; cyclic dinucleotides, CDNs.

Given its role as the inducer of IFN responses, it is unsurprising that the cGAS-STING pathway also holds significant importance in the development and proliferation of some tumors. For instance, a study highlights a notable connection between increased STING level and human papillomavirus-positive (HPV+) tongue squamous tumor [23]. Moreover, elevated STING expression is correlated with higher infiltration of regulatory T cells and release of interleukin-10 (IL-10) [24]. Both abnormal inflammation and STING deficiency are involved in tumor growth. Notably, STING deficiency in the inflammatory colitis-associated carcinoma murine model is linked to increased vulnerability to tumor development, whereas STING activation is correlated with accelerated tumor growth in the non-inflammatory mouse model of Lewis lung carcinoma [25]. Various elements of cGAS-STING could move across intercellular boundaries within the TME, enabling the transfer of cGAMP and pro-inflammatory cytokines among cells, thereby modulating the TME. These elements might exert opposing effects on tumor development [26].

Hence, the complex roles of the cGAS-STING pathway in tumors have been increasingly recognized, particularly in its dual functions of both promoting and suppressing tumor growth. This pathway not only influences tumor cell intrinsic mechanisms but also plays a significant role in modulating the TME. Building on this understanding, the roles of the cGAS-STING pathway in reprogramming TAMs have garnered significant attention. By activating the cGAS-STING pathway, TAMs can be reprogrammed from a pro-tumorigenic M2 phenotype to an anti-tumorigenic M1 phenotype, thereby enhancing the immune response against tumors. This reprogramming effect underscores the potential of targeting the cGAS-STING pathway as a therapeutic strategy to alter the TME and improve anti-tumor treatments.

## The roles of cGAS-STING pathway in reprogramming TAMs

### The functional complexity and reprogramming of TAMs in TME

As the most abundant immune cell population within TME, TAMs constitute approximately 50% in solid tumors [27]. TAMs are known to play critical roles in tumor growth and development [28]. The functional diversity and plasticity of TAMs enable TAMs to become promising targets for regulating the immune-suppressive TME and enhancing immunotherapy.

#### The functional complexity of TAMs in TME

Traditional dichotomy of TAMs includes M1 and M2 phenotypes. M1-like TAMs exhibit potent anti-tumor abilities and could produce elevated pro-inflammatory cytokines such as interleukin, thereby promoting inflammation and suppressing tumor growth. However, upon recruitment to tumor sites and exposure to anti-inflammatory cytokines in the TME, TAMs often adopt an M2 phenotype [29]. M2-like TAMs secrete abundant immunosuppressive chemokines and cytokines, effectively inhibiting immune responses such as antigen presentation and T cell functions, thereby promoting immunosuppression and tumor development [30]. Recent advancements using single-cell RNA sequencing (scRNA-seq) and spatial transcriptomics have revolutionized our understanding of the functional diversity of TAMs within TME and have revealed diverse TAM phenotypes that extend beyond the traditional M1/M2 dichotomy [31, 32]. For example, scRNA-seq studies in murine and human tumors have identified TAM subsets with unique transcriptional profiles associated with immune suppression, tissue remodeling, and even antigen presentation, highlighting their complex roles in tumor progression and immune evasion [31, 33]. Moreover, spatial transcriptomics has further illuminated the spatial distribution of TAM subsets within TME, showing how their functional states vary across diverse tumor regions. For example, TAMs in hypoxic regions often manifest immunosuppressive and pro-angiogenic abilities, while those in perivascular areas may support immune cell trafficking or contribute to vascular normalization [34, 35].

#### The reprogramming of TAMs in TME

The reprogramming of TAMs is dominated by both initial polarization state and the type and composition of stimuli in TME [36]. The diverse TAM functional states can be shaped by local stimuli, including cytokines (e.g., IL-10, TGF-β), metabolic stimuli (e.g., hypoxia, lactate), and interactions with tumor cells, stromal components, and other immune cells [37, 38]. And the dynamic plasticity and spatial heterogeneity of TAMs underscores their complex roles in tumorigenesis and immunotherapy, thus emphasizing the importance of considering the plasticity of TAMs and the physical and biochemical context of TME when designing therapeutic strategies [34, 35]. M1-like TAMs are associated with glycolysis and the release of free radicals [39]. These substances, highly cytotoxic in nature, play a crucial role in innate host defense and the initiation of pro-inflammatory responses, essential for the destruction of tumor cells. Conversely, IL-4 and IL-13 could induce TAM polarization towards M2 phenotype. M2-like TAMs activated by these cytokines engage the STAT6 signaling pathway, upregulate expression of mannose receptor-1 (CD206) and scavenger receptors (CD204, CD163), and secrete substantial amounts of anti-inflammatory factors, pro-angiogenic cytokines, and transforming growth factor-beta (TGF-β), thereby promoting a Th2 immune response [40]. Furthermore, M2-like TAMs are implicated in processes such as angiogenesis and tissue remodeling [41], consequently fostering tumor cell metastasis and intravasation, as well as contributing to therapeutic resistance and ineffective immune responses. This duality in function underscores the dual roles of TAMs in both innate and adaptive immunity. Given the unique functions and abundant presence of TAMs in many solid tumors, reprogramming TAMs from the M2 phenotype to the M1 phenotype is considered a promising strategy to disrupt the immune-suppressive TME and activate effective immune responses, thereby enhancing the efficacy of immunotherapy [15]. This approach holds the potential to overcome the limitations of current immunotherapies and serve as an effective strategy for anti-tumor treatment.

The high plasticity of macrophages enables TAMs to dynamically switch between phenotypes and functions in response to different stimuli and makes TAMs become promising targets for reversing the immune-suppressive TME and enhancing immunotherapy [42]. Accordingly, it is warranted to investigate the cGAS-STING pathway in TAMs, which is the most common antigen-presenting cells (APCs) in TME. It could help the application of STING agonists or antagonists in enhancing anti-tumor immunotherapy.

### The regulative roles of cGAS-STING pathway in TAMs

**Fig. 3:**
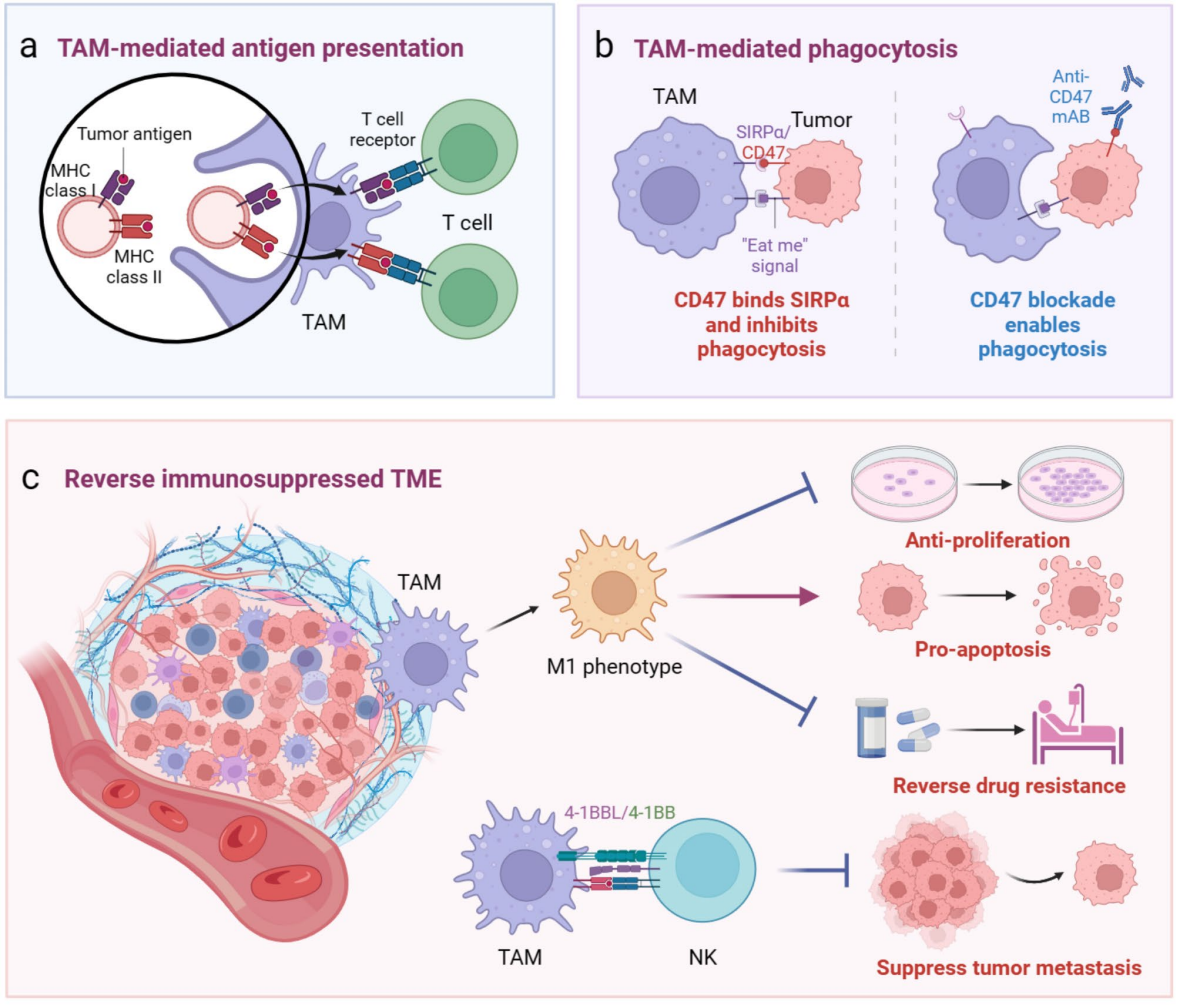
The regulative roles of cGAS-STING pathway in TAMs. (**a**) Regulating TAM-mediated antigen presentation via STING activation; (**b**) Modulation of TAM-mediated phagocytosis via STING activation; (**c**) Reprogramming TAMs via STING activation could reverse the immunosuppressed TME, enhance anti-proliferative and pro-apoptosis effects of TAMs, reverse chemotherapy resistance, and promote NK cell to suppress tumor metastasis

The intricate interplay among cytokines, chemokines, and immune cells in the TME significantly influences the efficacy of anti-tumor immunotherapy. STING administration has demonstrated therapeutic effects across various immune cells such as DCs, TAMs, NK cells, CD4 + T cells, and CD8 + T cells. Tumor-infiltrating DCs play a crucial role in capturing tumor-derived DNA via STING-based mechanisms, facilitating tumor-specific antigen presentation and activation of CTLs [43]. In the presence of STING agonists, NK cells contribute to eliminating tumors that are unresponsive to CD8 + T cells. Consequently, STING activation has the potential to induce a transformation in the TME [44], promoting its immunological sensibility and increasing the infiltration of other immune cells. This review mainly focuses on the regulative role of cGAS-STING in TAMs, which is the most common APC in TME (Fig. 3).

#### Regulating TAM-mediated antigen presentation via STING activation

As the APC in TME, TAMs could have the capability of antigen presentation to further activate anti-tumor immunity. Caiazza et al. [45] investigated the roles of STING in the TAM-mediated antigen presentation in the STING knockout macrophages. Owing to the knockout of STING, researchers observed compromised presentation of OVA-derived SIINFEKL peptide, coupled with reduced levels of MHC-I complexes on plasma membranes. This reduction might be attributable to downregulated mRNA expressions of the β2 m light chain. Furthermore, STING knockout influences the expression and phosphorylation of STAT1, which is pivotal for the formation of MHC-I-SIINFEKL complexes on plasma membranes, crucial for effective antigen presentations. These findings suggest a direct correlation between the absence of STING knockout and the hindered MHC-I antigen presentations by TAMs, mediated through β2-microglobulin inhibition and STAT1 activation.

#### Modulation of TAM-mediated phagocytosis via STING activation

Phagocytosis involves a multifaceted process that necessitates both the activation of pro-phagocytic “eat me” signals and the simultaneous disruption of anti-phagocytic “don’t eat me” signals [46]. Tumor cells utilize the “don’t eat me” signal transmembrane protein CD47 to evade phagocytosis. The CD47-signal regulatory protein alpha (SIRPα) pathway serves as a primary checkpoint for phagocytosis in TAMs. Targeting the CD47-SIRPα interactions to enhance TAM-mediated phagocytosis of tumor cells holds significant promise for treating various tumors [47]. Notably, anti-CD47 antibodies can bridge innate and adaptive immunity, with their efficacy requiring the presence of STING [48]. Dalton et al. [49] demonstrated that bone marrow (BM) macrophages inhibit acute myeloid leukemia through LC3-associated phagocytosis (LAP). Following the phagocytosis of apoptotic leukemic cells, including their mitochondria, mitochondrial DNA might activate STING pathway, contribute to inflammatory signals, enhance phagocytosis, and further inhibit leukemic cell proliferation. Based on the previous studies, Zou et al. [50] prepared the biomimetic nanovesicles named NAcp@CD47, capable of traversing the blood-brain barrier and delivering encapsulated anti-CD47 antibodies and STING agonist to glioblastoma (GBM) [51] to stimulate phagocytosis. The released anti-CD47 antibodies could reprogram M2 macrophages toward the M1 phenotype, augmenting GBM phagocytosis. Additionally, STING activation by CDG promotes IFN production, synergistically inducing a pro-inflammatory phenotype in TAMs and enhancing M1 macrophage-mediated phagocytosis of tumor cells, thereby significantly improving the efficacy of anti-CD47 blockade. These findings underscore the potential therapeutic utility of modulating TAM-mediated phagocytosis through STING activation in anti-tumor treatments.

#### Enhanced anti-proliferative and pro-apoptosis effects of TAMs via STING activation

It is reported that STING was inactivated in tumors including breast and lung carcinomas, with a noted positive association between STING expression and macrophage markers in these malignancies [52, 53]. Zhu et al. [54] discovered that vanillic acid (VA) can trigger the STING/TBK1/IRF3 pathway, leading to type I IFN production and promoting the M1 polarization, a process reliant on STING activation. Macrophages activated by VA-induced STING demonstrated anti-proliferative roles in tumor cells, although these effects were mitigated by STING antagonists and M2 macrophage-derived cytokines. More studies revealed that phagocytosis and apoptosis induction might be the primary mechanisms driving the anti-tumor effects of VA-treated macrophages. In details, VA enhanced macrophage polarization towards the M1 phenotype via IL-6R/JAK pathway, thereby amplifying phagocytosis and apoptosis-inducing capacities. Moreover, STING activation-induced IFN-β production contributed to apoptosis induction in tumor cells by VA-treated macrophages. It is suggested that enhanced anti-proliferative and pro-apoptosis effects of TAMs via STING activation could provide a new perspective for anti-tumor immunotherapy.

#### Reprogramming TAMs to reverse chemotherapy resistance and immunosuppressed TME

Numerous studies have highlighted the regulatory roles of TAMs in mediating chemotherapy resistance in tumors [55, 56]. Wang et al. [57] observed that TAMs attenuate the effectiveness of PARP inhibitors (PARPi). Mechanistically, in BRCA1-deficient breast tumor cells, TAMs might adopt the pro-tumor polarization state, suppressing PARPi-induced DNA damage in tumor cells. This suppression contributed to the decreased dsDNA fragments and synthetic lethality, ultimately inhibiting STING-dependent anti-tumor immune responses. STING agonist can reverse the polarization of M2-like macrophages, transforming them into the M1-like anti-tumor state via the STING-dependent manners. Systemic administration of STING agonists could overcome the tumor cell-induced immune suppression and synergize with PARPi to inhibit tumor development. Resistance to osimertinib, the next-generation EGFR-TKI, is also prevalent in clinical trials. Lin et al. [58] demonstrated that tumor inhibition induced by osimertinib relies on the activation of CD8 + T cells. Whereas, TAMs could accumulate in high-grade tumors hinder CD8 + T cell activation and diminish the response to osimertinib. Remarkably, reprogramming TAMs with STING agonist MSA-2 could revitalize anti-tumor immune responses and contribute to sustained tumor inhibition with osimertinib. Moreover, the anti-TGF-β/PD-L1 bispecific antibody YM101 has limited anti-tumor activity in immune-desert models. MSA-2 as a novel oral STING agonist, could activate the innate immune system and may synergize with YM101 in overcoming immunotherapy resistance [59].

Reprogramming TAMs via STING pathway activation could also reverse immunosuppressed TME. Shakfa et al. [60] investigated the potential of activating the STING pathway to enhance chemotherapy response and improve overall survival, particularly in high-grade ovarian tumors with PTEN deficiency. In vivo investigations revealed that Pten-deficient ovarian tumor cells show the immune-suppressed TME characterized by a higher presence of M2 macrophages and GR1 + MDSCs in ascites, along with impaired function of CD8 + T cells. When combined with chemotherapy, exogenous activation of STING led to prolonged survival in mice injected with Pten-deficient ID8 cells. Additionally, it transformed intraperitoneal M2 macrophages derived from Pten-deficient ascites into M1 macrophages and restored CD8 + T cell activation.

#### Macrophage STING signaling could promote NK cells to inhibit tumor metastasis

The innate immune response of macrophages plays a critical role in tumorigenesis, yet the roles and mechanisms of macrophage STING signaling in remodulating TME to suppress tumor remain largely unknown. Sun et al. [61] discovered that macrophage STING signaling could promote NK cell-mediated suppression of liver metastasis in colorectal cancer (CRC) through 4-1BBL/4-1BB co-stimulation. They observed upregulated activation of STING pathway in tumor tissues, as well as in intrahepatic macrophages. STING activation led to IL-18 and IL-1β secretion of macrophages, which enhanced the anti-tumor capability of NK cell by inducing 4-1BBL expression in macrophage and 4-1BB in NK cell, respectively. Furthermore, treatment with MC38 activated macrophage NLRP3 signaling, which was suppressed by STING depletion. Deficiency of myeloid NLRP3 promoted tumor burden and inhibited NK cell activation. These findings demonstrate that STING can enhance NLRP3-induced production of IL-18 and IL-1β by macrophages, thereby optimizing the anti-tumor effects of NK cell through 4-1BBL/4-1BB co-stimulation.

#### Crosstalk with other signaling pathways in regulating STING of TAMs

While STING-Type I IFN signaling in TAMs is of great importance in effective anti-tumor immunity, STING agonists exhibit limited efficacy as monotherapy in clinical trials. Ho et al. [62] revealed that protein phosphatase 2 A (PP2A), along with its specific B regulatory subunit Striatin 4 (STRN4), exerted negative regulation on STING-Type I IFN in macrophages. STRN4 is reported to regulate the Hippo-Yes-associated protein (Hippo-YAP) pathway. They reported that the Hippo kinase mammalian STE20-like protein kinase (MST1/2), a negative regulator of YAP and a transcriptional coactivator with PDZ-binding motif (TAZ), is essential for STING activation. STRN4 associated with PP2Ac to dephosphorylate MST1/2, consequently stabilizing YAP/TAZ and antagonizing STING activation. As for GBM, YAP/TAZ expression was notably high in TAMs but not in non-tumor macrophages. Furthermore, PP2A/STRN4 deficiency in macrophages reduced YAP/TAZ expression and sensitized TAMs to STING stimulation. Thus, the PP2A/STRN4-Hippo-YAP/TAZ signaling axis plays a crucial role in regulating STING-Type I IFN in TAMs. This study underscores the potential for targeting this pathway to promote anti-tumor immunity through combination with other STING-activating strategies.

Oxidative stress-induced ferroptosis and inflammation mediated by macrophages play pivotal roles in various liver diseases. In their study, Su et al. [63] investigated how hepatocyte ferroptosis regulates macrophage STING activation in the progression of spontaneous liver damage, fibrosis, and tumorigenesis, using a model induced by deficiency of transforming growth factor-beta-activated kinase 1 (TAK1). The study revealed that oxidative DNA damage resulting from hepatocellular ferroptosis could promote macrophage STING activation, thereby facilitating liver diseases. Inhibition of ferroptosis with ferrostatin-1 attenuated macrophage STING activation, resulting in reduced liver injury, fibrosis, and tumor burden. Mechanistically, elevated levels of 8-hydroxydeoxyguanosine were shown in the liver and serum of TAK1-deficient mice, which were prevented by ferroptosis inhibition. Treatments with an antibody against 8-hydroxydeoxyguanosine could inhibit macrophage STING activation in TAK1-deficient mice.

To sum up, both Hippo-YAP/TAZ signaling and oxidative stress-mediated ferroptosis could regulate the STING signaling of TAMs. In addition to activating STING signaling directly, we have more choices to highlight the crosstalk with other signaling pathways in regulating macrophage STING activation.

## Targeting cGAS-STING pathway for reprogramming TAMs

The roles of the cGAS-STING pathway in reprogramming TAMs have garnered significant attention, as it offers a promising avenue to modulate the TME. By activating this pathway, TAMs can be shifted from a pro-tumorigenic M2 phenotype to an anti-tumorigenic M1 phenotype, enhancing immune-mediated tumor suppression. This reprogramming capability highlights the critical influence of the cGAS-STING pathway on immune cell within tumors.

Given its pivotal role in reshaping TAMs, targeting the cGAS-STING pathway has emerged as a compelling therapeutic strategy. Researchers are exploring various approaches, such as STING agonists or cGAS activators, to harness this pathway’s potential for reprogramming TAMs. These efforts aim to create a more favorable anti-tumor immune environment, potentially improving the efficacy of existing anti-tumor treatments and paving the way for novel immunotherapies.

### Traditional cGAS-STING pathway modulators

As the main target in immunotherapy, the cGAS-STING pathway could promote innate and specific immunity and exhibit anti-tumor effects [64, 65]. Traditional cGAS-STING pathway modulators include the STING agonists and antagonists. The clinical trials in tumors focusing on cGAS-STING pathway modulators are concluded in Table 2.

**Table 2:** Clinical trials in tumors focusing on cGAS-STING pathway modulators

Agent	Target	Mechanism	Tumor	Notes	Citations
**ADU-S100**	STING	Binding with LBD in STING	Advanced/metastatic solid tumors or lymphomas	Evaluated as a monotherapy and in combination with ICIs	NCT03172936
**MK-1454**	STING	Binding with LBD in STING	Advanced/metastatic solid tumors or lymphomas	Assessed alone and with ICIs such as pembrolizumab	[96] NCT04220866
**BMS-986,301**	STING	STING agonist	Advanced solid tumors	In combination with nivolumab	NCT03956680
**Suramin**	cGAS	The inhibition of cGAS enzymatic activity	Stage IIIB-IV breast cancer	Combined with paclitaxel	NCT00054028
**Aspirin**	cGAS	Acetylated Lys amino group of cGAS protein	Colorectal cancer	Explored for its potential to modulate the cGAS-STING pathway, aiming to reduce platelet activity and inflammation	NCT03603366
**DMXAA**	STING	Binding with m-STING	Solid tumors, advanced non-small cell lung cancer	Initially showed promise in preclinical models but failed to demonstrate efficacy in human trials due to species-specific STING activation	[97]

**Abbreviations:** cyclic GMP-AMP synthase, cGAS; stimulator interferon genes, STING; immune checkpoint inhibitors, ICIs.

#### The STING agonists in the treatment of tumors

It is widely recognized that STING agonists, which stimulate the cGAS-STING-IFN pathway, are effective in triggering systemic pro-inflammatory responses to eliminate tumors. Given the pivotal role of cGAS-STING in innate immune responses and various pathological conditions, there has been a surge in the development of pharmacological agonists targeting STING. STING agonists include cyclic dinucleotide STING agonists, non-nucleotide, small molecule STING agonists and cGAS agonists [22]. Cyclic dinucleotide STING agonist includes natural cyclic dinucleotides (CDNs) as well as derivatives based on native CDN structures [66]. Whereas, the negative charge, hydrophilicity, and instability of CDNs limit their cytoplasmic delivery efficiency and render them unsuitable for clinical application. Certain chemically modified CDN derivatives with enhanced lipophilicity and stability exhibit improved medicinal potential, although most are still in pre-clinical trials. Non-nucleotide small molecule STING activators exhibit superior pharmacological properties and could more efficiently penetrate the cytosol. One study compared the anti-tumor efficacy between intratumoral and intramuscular systemic administration of BMS-986,301, a next-generation STING agonist. This study demonstrated that administration of STING agonist systemically via intramuscular injection is equivalent to its intratumoral injection in inducing both effector T cell response and antitumor efficacy, which supports the clinical development of STING agonists via systemic administration for treating tumors [67].

STING agonists hold promise as adjuvants for immune-checkpoint blockade (ICB) therapy and radiotherapy [68, 69]. Consequently, pro-clinical trials have been performed to assess the therapeutic effects or potential adverse effects of STING agonists, either alone or in combination with ICB [70–72]. However, emerging evidence suggests that the efficacy of STING agonists may diminish at high dosages when administered locally. Furthermore, their broader application in vivo is impeded by the instability of cytoplasmic DNA [73].

#### The STING antagonists in the treatment of tumors

STING antagonists, including covalent STING inhibitors, may also exhibit anti-cancer effectiveness [74]. At present, while genomic and phenotypic indicators of chromosomal instability have been recognized to differentiate patients who could potentially gain from STING inhibition or activation, it is suggested that additional research and pre-clinical trials should be conducted to clarify the utility of STING agonists or antagonists across different tumor types or stages [75].

The correlation between cGAS-STING activation and tumor metastasis has spurred further assessment and exploration of STING agonists in tumor treatment, highlighting the intricate roles of cGAS-STING in tumors. This observation presents a dual perspective: it suggests the potential and rationale for utilizing STING antagonists in advanced-stage tumors, while also prompting the investigations of reconsidering the potential adverse effects of STING agonists in anti-tumor treatment. In details, tumor cells with chromosomal instability often harbor micronuclei, which chronically activate cGAS-STING and use STING for survival [76, 77]. This mechanistic insight underscores the rationale for utilizing STING antagonists in advanced-stage tumors.

### The strategies of targeting cGAS-STING for reprogramming TAMs

**Fig. 4:**
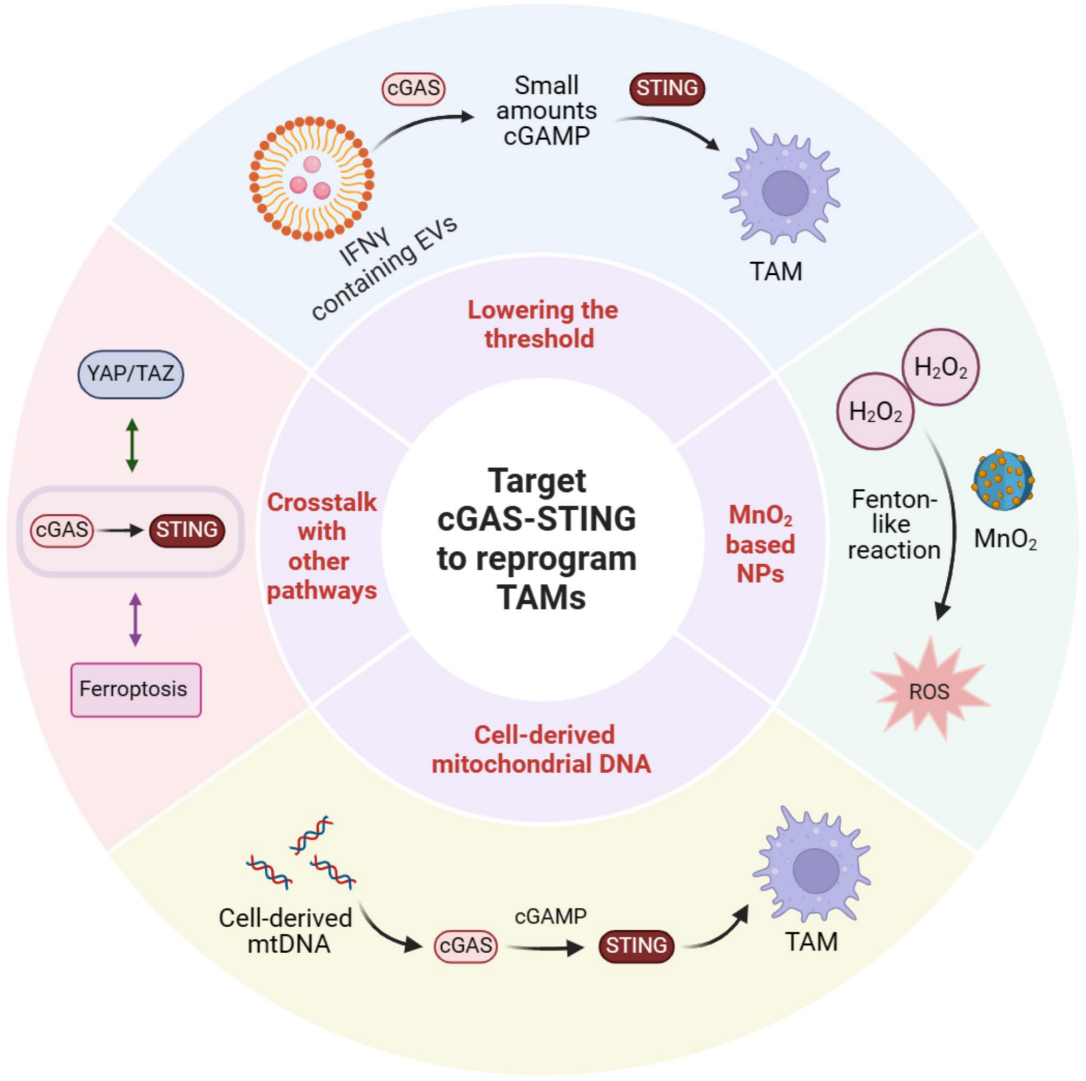
The strategies of targeting cGAS-STING for reprogramming TAMs. Several strategies of targeting cGAS-STING for reprogramming TAMs are warranted for enhancing anti-tumor immunotherapy. For instance, some novel strategies have been applied in anti-tumor immunotherapy: lowering the threshold for STING activation in TAMs; preparing MnO_2_ based NPs for regulating STING activation; cell-derived mtDNA inducing STING activation for resetting anti-tumor immunity; crosstalk with other signaling pathways in regulating STING of TAMs

Activation of the STING pathway in the TME has shown great anti-tumor responses in pre-clinical trials. Whereas, these inspiring effects have not been achieved in clinical applications to date [78]. The utilization of STING agonists as monotherapy or in conjunction with ICB has shown limited success in yielding partial or complete responders. It could potentially suggest that the dosage of STING agonists is either insufficient to induce signals or excessive, leading to the depletion of crucial immune cells within the TME necessary for eliminating tumor cells [79]. Therefore, more strategies of targeting cGAS-STING for reprogramming TAMs are warranted for enhancing anti-tumor immunotherapy (Fig. 4).

#### Lowering the threshold for STING activation in TAMs

To determine the optimal dosage of STING agonists for clinical use, it is imperative to develop strategies that widen the therapeutic window to effectively activate STING. One possible reason why STING agonists fail to elicit a robust anti-tumor response is the high physiological threshold required for activating STING of immune cells [80]. The key problem arising from it might be the expected endogenous levels of cGAMP in the TME. In the study of Hansen et al. [81], they elucidated a promising strategy to lower the threshold for activating STING of immune cells. They demonstrated that extracellular vesicles (EVs) derived from activated CD4 + T cells could prime macrophages and enhance their responsiveness to minimal amounts of cGAMP. The priming mechanisms might contribute to the rapid and robust cytokine induction profiles, sensitizing tumors to effectively control tumor development at suboptimal doses of STING agonists. Their findings suggested that IFNγ containing EVs derived from activated CD4 + T cells could sensitize macrophages to STING. Consequently, these EVs had the potential to remodel the immune-suppressive TME by regulating macrophage polarization. Moreover, the EVs could increase the sensitivity of immune cells to low concentrations of STING agonists within the TME, thereby supporting STING activation. Despite the uncertain outcomes of clinical trials investigating STING agonists as anti-tumor treatments, active clinical development persists [82]. EVs from activated immune cells might represent a promising novel avenue for therapy, particularly in combination with ICB or other treatments that promote increased intratumoral cGAMP production, such as radiotherapy or chemotherapy.

#### Manganese oxide (MnO2) based nanoparticles (NPs) for regulating STING activation

Clinical trials involving STING agonists have demonstrated limited anti-tumor effects and dose-dependent adverse effects, such as inflammatory damage and cellular toxicity. Metal ions such as Mn^2+^ might be involved in the Fenton-like reactions, generating hydroxyl radical (•OH) from endogenous H_2_O_2_ [83, 84]. These radicals could induce oxidative stress in tumor cells while simultaneously promoting TAMs polarization into M1 phenotypes. Furthermore, Mn^2+^ has been shown to enhance the activation of STING [85, 86], thereby holding significant promise for enhancing anti-tumor immune responses. For example, Mn^2+^ could activate STING pathway and promote the maturation of human and murine DC. One previous study showed that Mn^2+^ plus anti-TGF-β/PD-L1 bispecific antibody YM101 treatment had a more powerful antitumor effect and a broader antitumor spectrum [87]. However, the administration of free Mn^2+^ may have side effects on normal tissues. To address this issue, MnO_2_ nanosheets or NPs with properties that enable degradation in response to H^+^/glutathione (GSH) in the TME have been proposed [88]. These nanomaterials could offer a more suitable approach for reprogramming the TME and facilitating synergistic therapy.

Zhang et al. [89] constructed the versatile biomimetic NPs termed as HM-BPT, utilizing pH-sensitive tumor-tropism hybrid membrane-coated MnO_2_ NPs for the delivery of BPTES, a glutamine metabolism inhibitor. Essentially, the hybrid membranes composed of mesenchymal stem cell membranes (MSCm) and pH-sensitive liposomes (pSL) allowed the biomimetic NPs to target the TME and reduce endocytosis. It revealed that treatment with HM-BPT contributed to significant tumor suppression, CTL infiltration, and macrophage polarization towards an M1 phenotype and activation of the STING pathway in a 4T1 xenograft model. Additionally, synergistic depletion of GSH and oxygen (O_2_) supply ameliorated the immune-suppressive TME, enhancing anti-tumor immunity. Due to the low pH and high GSH concentration in the TME, HM-BPT NPs were degraded to release BPTES while simultaneously producing Mn^2+^ and O_2_. It resulted in the increased CTL infiltration within tumors, while the abundance of immune-suppressive cells including regulatory T cells (Tregs) was significantly reduced. Furthermore, intratumoral macrophages showed the substantially increased M2 phenotypes, along with robust STING activation when treated with HM-BPT. This robust immune response contributed to significant tumor inhibition.

Tetrahedral DNA nanostructures (TDNs) have demonstrated exceptional potential in the fields of drug delivery and biomedical treatment [90]. Liang et al. [91] demonstrated that TDNs actively penetrated macrophages, leading to the promotion of STING activation and M1 polarization in a size-dependent way. They also found that TDNs synergized with Mn^2+^ to increase IFN-β expression, inducible nitric oxide synthase (iNOS), and co-stimulatory molecule involved in antigen presentations. Furthermore, to mitigate the cytotoxicity associated with Mn^2+^, they developed a TDN-MnO_2_ complex, which exhibited significantly higher efficacy compared to TDNs combined with Mn^2+^ alone in initiating macrophage activation and eliciting anti-tumor responses. Overall, their study elucidated a significant immune response of TDNs in anti-tumor treatments and highlighted their synergistic therapeutic potential when combined with MnO_2_.

#### Cell-derived mitochondrial DNA inducing STING activation for resetting anti-tumor immunity

Mitochondrial damage-associated molecular patterns (mtDAMPs) encompass proteins, lipids, metabolites, and DNA, exhibiting several immune-regulatory functions [92]. Among these, cell-derived mitochondrial DNA (mtDNA) stands out as the activator of innate immune systems, serving as a type of mtDAMP [93]. In the context of multiple myeloma (MM), the interactions among cells in the bone marrow (BM) microenvironments are important in disease survival and progression. Jibril et al. [93] elucidated the roles of MM cell-derived mtDAMPs in shaping the BM microenvironments and described the functional consequences of mtDAMPs in MM development. Their study revealed that BM macrophage could sense and respond to mtDAMPs via STING, and inhibition of STING resulted in a reduction of MM tumor burden. Furthermore, MM-derived mtDAMPs could induce the increase of chemokine signatures in BM macrophage, and inhibition of it might lead to the egress of MM cells from the BM. In summary, tumor cells release mtDNA, a form of mtDAMP, into the BM microenvironments, thereby activating macrophages via STING signaling.

Plant-derived nanovesicles (PDNVs) have emerged as the significant mechanisms for inter-kingdom interactions [94]. However, the specific effector components within these NVs and their underlying mechanisms remain elusive. Liu et al. [95] undertook the isolation and purification of exosome-like NPs from Artemisia annua, which are nano sized and membrane-bound shaped as artemisia-derived nanovesicles (ADNVs). They could identify plant-derived mtDNA as a major effector molecule encapsulated within these NVs. Upon internalization into TAMs via the NVs, plant-derived mtDNA induced cGAS-STING, promoting the conversion of anti-tumor macrophages. Moreover, ADNVs significantly enhanced the effects of programmed death-ligand 1 (PD-L1) inhibitors in tumor-bearing mice. This study sheds light on an intriguing the inter-kingdom interaction, where plant-derived mtDNA delivered by NVs, stimulates immunostimulatory signaling in mammalian immune cells. This phenomenon might play a pivotal role in resetting anti-tumor immune response and facilitating tumor eradication.

## Future perspective and conclusion

As for the development of tumors, several mechanisms including unresolved pathological inflammation combined with disrupted metabolism exist. TAMs are of great importance in tumor immunotherapy, influencing both innate and specific immunity. As the major infiltrating cells in TME, TAMs could be polarized into either M1 or M2 types based on various stimuli. Accordingly, targeted manipulation of TAMs to restore immune balance shows promise as an effective anti-tumor strategy. Whereas, there is no doubt that cGAS and STING have distinct and independent functions. cGAS-STING pathway is pivotal in activating both innate and specific immunity within anti-tumor immunotherapy. Monocytes, particularly macrophages, serve as the main source of type 1 IFN through this pathway in human body. Thus, investigating the roles of cGAS-STING in TAMs and its effects on the TME are of great importance. This review aims to target the cGAS-STING to reprogram TAMs, thereby enhancing anti-tumor immunotherapy.

Many researches have paid special attention on the double-edged sword effects of cGAS-STING pathway in tumors. The cGAS-STING might generate IFN, promote the maturation and migration of DCs, activate TAMs and NK cells, and induce the expression of chemokines for the infiltration of CTLs. The above processes induce the anti-tumor immune response [21]. Considering its function as an inducer of IFN responses, it is not surprising that cGAS-STING also could exert a significant influence on the development and proliferation of some tumors. Different elements of cGAS-STING are capable of crossing the intercellular boundaries in TME, enabling the transfer of cGAMP and pro-inflammatory cytokine among cells, thus influencing the TME. These elements might exert opposing effects on tumor progression [26]. Hence, there is a need for further investigation into the double-edged sword effects of cGAS-STING in regulating TME. Based on the above effects, it is necessary to investigate the cGAS-STING in TAMs, which is the most common APCs in TME. It could help the applications of STING agonists or antagonists for enhancing anti-tumor immunotherapy.

Our review concluded the regulative effects of cGAS-STING in TAMs as follows: (a) regulating TAM-mediated antigen presentation via STING activation; (b) modulation of TAM-mediated phagocytosis via STING activation; (c) enhanced anti-proliferative and pro-apoptosis effects of TAMs via STING activation; (d) reprogramming TAMs to reverse chemotherapy resistance and immunosuppressed TME; (e) macrophage STING signaling could promote NK cells to inhibit tumor metastasis. It highlights the pivotal roles of cGAS-STING in regulating the functions of TAMs, which builds a foundation of targeting cGAS-STING pathway for reprogramming TAMs.

More importantly, several strategies of targeting cGAS-STING for reprogramming TAMs are warranted for enhancing anti-tumor immunotherapy. For instance, some novel strategies have been applied in anti-tumor immunotherapy: (a) lowering the threshold for STING activation in TAMs; (b) preparing MnO_2_ based NPs for regulating STING activation; (c) cell-derived mtDNA inducing STING activation for resetting anti-tumor immunity; (d) crosstalk with other signaling pathways in regulating STING of TAMs.

In conclusion, although the developments of cGAS-STING agonists and antagonists is still in its early stages, we anticipate further basic research and pre-clinical trials aimed at targeting the cGAS-STING for reprogramming TAMs. This endeavor holds great significance in the realm of anti-tumor immunotherapy.

## Data Availability

No datasets were generated or analysed during the current study.
